# Out of the blue: understanding abrupt and wayward transitions in thought using probability and predictive processing

**DOI:** 10.1098/rstb.2019.0692

**Published:** 2020-12-14

**Authors:** Caitlin Mills, Andre Zamani, Rebecca White, Kalina Christoff

**Affiliations:** 1 Department of Psychology, University of New Hampshire, Durham, NH, USA; 2 Department of Psychology, University of British Columbia, Vancouver, British Columbia, Canada; 3 Centre for Brain Health, University of British Columbia, Vancouver, British Columbia, Canada; 4 Peter Wall Institute for Advanced Studies, University of British Columbia, Vancouver, British Columbia, Canada

**Keywords:** spontaneous thought, thought transitions, mind-wandering, predictive processing, phenomenology

## Abstract

Thoughts that appear to come to us ‘out of the blue’ or ‘out of nowhere’ are a familiar aspect of mental experience. Such thoughts tend to elicit feelings of surprise and spontaneity. Although we are beginning to understand the neural processes that underlie the arising of such thoughts, little is known about what accounts for their peculiar phenomenology. Here, we focus on one central aspect of this phenomenology—the experience of surprise at their occurrence, as it relates to internal probabilistic predictions regarding mental states. We introduce a distinction between two phenomenologically different types of transitions in thought content: (i) *abrupt transitions*, which occur at surprising times but lead to unsurprising thought content, and (ii) *wayward transitions*, which occur at surprising times and also lead to surprising thought content. We examine these two types of transitions using a novel approach that combines probabilistic and predictive processing concepts and principles. We employ two different probability metrics—transition and occurrence probability—to characterize and differentiate between abrupt and wayward transitions. We close by discussing some potentially beneficial ways in which these two kinds of transitions in thought content may contribute to mental function, and how they may be implemented at the neural level.

This article is part of the theme issue ‘Offline perception: voluntary and spontaneous perceptual experiences without matching external stimulation’.

## 1. Introduction

There are many colloquial expressions that are used to describe the peculiar experience of a thought catching us by surprise as it surfaces into awareness. Such thoughts are accompanied by a feeling of spontaneity in their arising and appear to come ‘out of the blue’ or ‘out of nowhere’. This compelling sense of out-of-nowhere-ness arises because we cannot immediately tell, at the subjective level, why this thought with its particular contents should have occurred at that particular moment of time. This experience is quite distinct from the many other thoughts we experience, whose sources are readily traceable to something in the preceding stream of thought or our environment [1–3].

Such surprising, spontaneity-eliciting thoughts are an important and familiar feature of our mental experience. Although we are beginning to understand the neural processes that underlie the arising of such thoughts [4–6], we know relatively little about the sources behind their phenomenology: why do we experience these mental arisings as surprising and spontaneous, and what makes these thoughts (but not others) elicit those feelings?

Here, we offer a close examination of this out-of-nowhere-ness phenomenology in an attempt to answer these questions. Although such thoughts may be related to other mental phenomena such as mind-wandering, we do not conflate these complex and mutually interrelated phenomena. Mind-wandering encompasses a broad range of mental experiences that can include mental content with a readily identifiable stimulus-related [5,7] or goal-related [8] source, and do not necessarily elicit feelings of surprise or spontaneity. A detailed discussion of mind-wandering and its relationship to the spontaneous thought is outside the scope of the current paper but can be found elsewhere [5,9–11].

### (a) The experience of spontaneity

Let us start by considering two different phenomenological experiences that are examples of surprise-inducing thought transitions. First, imagine the following scenario. You are listening to a lecture when suddenly your attention is captured by an unexpected thought: you wonder if your dog has been alone at home for too long that day. This thought has arisen without any deliberate intention on your part, you did not anticipate its arising and you wonder why you should think of this at that particular time. Here, we refer to this change in thought content as *abrupt transition*—the arising of a thought that is not deliberately generated but nonetheless has an elevated probability of occurrence owing to underlying affects, motivations, goals or current concerns [12–14]. The fact that you care about your dog makes thoughts about your dog occur with increased frequency, although you cannot necessarily predict exactly *when* they will arise. The moment when the transition occurs is surprising, but the content of the thought is not.

Now, imagine a second scenario. You are listening to the same lecture when you suddenly find yourself thinking about a playground you used to play at in your childhood. Like the example above, this thought is not deliberate and feels unexpected. However, even though you try, you cannot see any reason why you should think about this at that particular point. You do not have any strong feelings about this playground, and you have not thought about it for decades. We refer to this change in thought content as *wayward transition*.^1^ The moment the transition occurs is surprising and so is the content it leads to.

There is a convergence between abrupt and wayward transitions at the phenomenological level: both elicit the experience of surprise and are often experienced as spontaneous mental events. One cannot predict exactly when either type of thought transition will occur. They are both also likely to be unintentional and task-unrelated—a commonly used but debated [9–11] definition of mind-wandering.

There is also, however, a key point of *divergence* between the two types of transitions: while abrupt transitions may surprise us with the timing of their occurrence, their content is not surprising once we reflect on its close connection to our emotions, motivations, goals and current concerns. By contrast, wayward transitions cannot be explained by any obvious affect, motivations, implicit goals or current concerns: the content of the thought itself is surprising, as well as the timing of its occurrence. Wayward transitions may often take the form of involuntary semantic memories—when words, images and other mental contents come to mind unexpectedly and often without any identifiable triggers [15]. At other times, wayward transitions may arise in the form of involuntary episodic memories—when past events come to mind without an attempt at retrieval [16]. Although involuntary episodic memories are generally more likely to have identifiable sources [2], they may induce surprise and spontaneity if such identifiable sources are lacking.

Because wayward transitions are more likely to feel puzzling in their origins than abrupt transitions, they may also elicit a stronger feeling of surprise and spontaneity. It is worth noting, however, that neither of these two kinds of transitions is likely to be a truly ‘spontaneous’ event at the physiological level: neural processes below the level of conscious awareness may well explain their arisings, as neuroimaging findings are beginning to suggest [4–6]. Nevertheless, both wayward and abrupt transitions appear to be experienced as spontaneous at the phenomenological level and it is important to account for this phenomenological reality in our scientific accounts of mental spontaneity.

Here, we propose that the phenomenological differences between abrupt and wayward transitions may be accounted for by adopting a probability-based approach. In what follows, we first describe the application of probabilistic and predictive processing concepts and principles to understanding the phenomenology of spontaneity in thought, including how implicit statistical models may predict thought content and transitions. We also describe two different probability metrics—transition and occurrence probability—that are likely computed as part of those implicit statistical models. We then propose that these two probability metrics could help characterize and distinguish between abrupt and wayward transitions. Finally, we discuss some potentially beneficial ways in which abrupt and wayward transitions may contribute to mental function, and how they may be implemented at the neural level.

## 2. Statistical models of thought

The experience of surprise can be viewed as stemming from *a breach of expectation*. Such breaches of expectation are a central feature in multiple well-validated predictive processing accounts of exteroceptive and interoceptive processing [17–21]. In this section, we use explanatory concepts from the predictive processing literature in an attempt to examine more closely the distinction between abrupt and wayward transitions, and to better understand how and why some thoughts elicit a feeling of surprise as they arise.

### (a) Predictive processing overview

Predictive processing posits that instead of passively receiving and interpreting the sensory and non-sensory signals it receives, the brain processes those signals actively by continuously issuing proactive predictions about what the incoming signals in each next moment will be. These predictions are formed through the brain's modelling of statistical regularities in the world. Through the issuing of these predictions, all incoming information can be processed as a match or a mismatch relative to the predictions. Predictive processing allows the brain to process the multitude of incoming signals highly efficiently, by transmitting upwards into the neural hierarchies only the unpredicted portions of these signals (the mismatches) and filtering out the predicted portions (the matches). The mismatches are known as prediction errors. Prediction errors can be used to update future predictions by propagating prediction error signals upward through the neural hierarchies [17]. For a comprehensive and in-depth discussion of predictive processing, see [20,22–24]. Predictive processing—including the computation of implicit statistical models, the issuing of predictions and the generation of prediction error—is generally assumed to operate outside of conscious awareness (‘consciousness’ defined as pure phenomenological experience [25]), even though its effects may have an upstream conscious effect [23].

There is ample evidence that such predictive processing principles operate throughout our everyday lives. Imagine that you have just been served coffee; you take a sip expecting it to be hot, but instead it is cold. You only realize your brain had issued a prediction that the coffee would be hot once your brain generated the prediction error resulting from the violation of that expectation. You can now consciously become aware of the implicit prediction about the temperature of served coffee that your brain has formed through past experience.

We propose that these predictive processing principles can also be applied to understand how the brain constructs implicit statistical models and predictions about various aspects of our own thought stream. This would include implicit models capturing the statistical regularities in the kinds of mental transitions we tend to experience, the types of things we tend to think about and the frequency with which we tend to think about them. Although in the discussion below, we sometimes refer to ‘statistical model of thought’ in the singular, we acknowledge that it is likely not a singular mechanism.

To our knowledge, there have been two other attempts so far to examine the usefulness of statistical models at the level of thought [26,27]. These pioneering accounts proposed functions for spontaneous thought that relate to predictive processing, including active inference [26] and event models [27]. They did not, however, address how thoughts themselves may be incorporated into implicit statistical models or how the violation of predictions about what our next thought will be can contribute to experiencing that next thought as surprising or spontaneously arising. Our account specifically addresses these latter points by proposing how statistical regularities in the thought stream may shape forward thought predictions and may contribute to the phenomenology of surprise and spontaneity in thought.

### (b) Modelling statistical regularities in thought

Thoughts are an inseparable part of conscious experience and arise frequently [4,28] so much so that they sometimes appear to form a continuous, uninterrupted ‘stream of consciousness’ [29]. This ‘stream’ makes up a vast portion of our mental experience throughout most of the lifespan, providing ample opportunities for extracting statistical regularities of its various features, including different thought transitions and content frequencies, in order to form predictions about the ‘stream’.

Many of our thoughts arise in congruence with the brain's predictions for their contents and manner of arising, leading to a low degree of prediction error. Such thoughts come and go without a feeling of surprise, and are often easily attributable to elements in our internal or external environment that act as triggering cues [1–3]. Some thoughts, however, do generate a high degree of prediction error as they arise. We propose that the feeling of surprise and spontaneity are phenomenological correlates of such prediction errors in thought. We also propose that different types of prediction errors may help characterize and distinguish between abrupt and wayward transitions. Both types of transitions would be associated with prediction error as to *when a thought should occur*. Only wayward transitions, however, would be associated with prediction error as to *whether a thought should occur at all*. These predictions and prediction error computations likely operate at largely unconscious levels, and may underlie the peculiar phenomenology of surprise at one's own thoughts.

**Figure 1.**
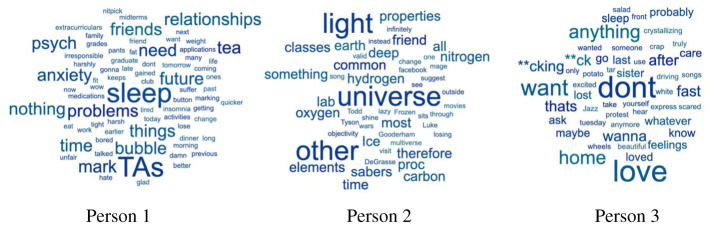
Variability in thought content across individuals. Word font size indicates the frequency of each word's occurrence in each person's thought report. (Online version in colour.)

The degree to which different thoughts generate prediction error would be unique to each person. Different individuals have different statistical regularities in the occurrence of their thought contents and would, therefore, have different implicit statistical models of their own thought streams. The word clouds in figure 1 come from an unpublished dataset from our laboratories and illustrate this variability across different individuals. These word clouds are from three different individuals who participated in a modified think-aloud task. During this task, individuals sat at a computer with a blank screen in front of them and were told they could think about whatever they wanted. Every 2–3 min during the 30 min experimental session, they reported their most recent thought stream by typing on the computer in front of them. The word clouds show the 50 most frequently occurring content words in each individual's thought streams. Each word's font size corresponds to the number of times this word occurred in the participant's report (e.g. ‘sleep’, 3 times; ‘universe’, 4 times).

There are marked differences in thought content and frequency among these three individuals' reported thought contents. For example, Person 1 appears much more likely to think about worries such as marking, sleep, anxiety and relationships; Person 2, on the other hand, is much more likely to think about scientific concepts such as the Universe, light, carbon and hydrogen. High probability thought content for one person would appear quite unsurprising to that person. If the same thought content occurred to another person, however, it is likely to be experienced as a surprising wayward transition—for example, if Person 1 suddenly had a thought about the Universe and its nitrogen, hydrogen and carbon contents. On the other hand, each person may experience the occurrence of any of their own high probability thought contents as an abrupt transition, if it occurs at an unexpected time. Although these data provide a very limited window into these three individuals' streams of thought, they clearly demonstrate the high degree of variability in thought content across individuals.

In all likelihood, the implicit models of thought's statistical regularities span multiple timescales, serving to incorporate different contexts into predictions. In the case of an acutely stressful life event—for example, a family health problem—the statistical models would be updated so that thought content related to the health concern will have a higher predicted likelihood of occurring, with a corresponding reduced degree of prediction error generated at its onset.

### (c) A probabilistic approach: transition versus occurrence probability

The distinction between abrupt and wayward transitions can be further probed by using specific probability metrics to answer the following questions with respect to a given thought: first, what was the probability (i.e. the predicted likelihood) that this thought would occur *at this particular moment*? And second, what was the probability that this thought would occur *at all*? These questions underlie two different probability metrics: transition and occurrence probability, respectively. The distinction between transition and occurrence probability has been discussed extensively elsewhere in the context of exteroceptive processing [30–32]. Here, we discuss its relevance and utility for understanding abrupt and wayward transitions in thought content.

*Transition probability* refers to the probability that a given thought, with its particular content and features, will occur, given that another thought with its own specific content and features has just occurred. In more technical terms, it is the conditional likelihood that thought *n* *+* 1 will occur, given that thought *n* has just occurred [33,34], or *P*(*n* *+* 1|*n*). The lower the transition probability of thought *n* *+* 1 is, the greater the feeling of surprise would be during its arising. Transition probabilities (also known as ‘transitional probabilities') are often used to describe implicit statistical learning processes in language development. This implicit learning is thought to occur through establishing a probabilistic model reflecting one's experience with words and sounds over time [34,35]. The basic idea is that language, perhaps much like our thoughts, has a probabilistic nature: spoken utterances are rarely unpredictable, and often word *n* *+* 1 in a sentence will have some conditional dependency on word *n*. Learners, especially infants, develop implicit models of such dependencies by tracking statistics like overall frequencies and co-occurrence of sounds and words, as well as physical and social context, among other possible cues [33,35]. The statistical regularities of such cues provide information to the learner about language and grammar structure in addition to word meanings.

*Occurrence probability*, on the other hand, refers to the probability that a thought will arise based upon its overall baseline frequency. It is another form of conditional probability: the conditional likelihood that a thought will arise, given the overall baseline frequency of that thought occurring at any point in the past. As the word clouds in figure 1 suggest, baseline frequencies differ across different thought contents within the same individual, as well as across individuals (someone may think about their teaching assistantship duties 10% of the time, whereas another person may never think about this). Thoughts relating to current concerns, life goals, emotions and habits (recall the scenario of spontaneously thinking about one's dog during a lecture) would have higher occurrence probability. Conversely, thoughts that have lower baseline frequency would have lower occurrence probability. Such low occurrence probability thoughts may take the form of spatio-temporally distant memories with low personal significance, such as the earlier example of a childhood playground.

By considering the two metrics of transition and occurrence probability jointly, it may be possible to better characterize and distinguish between abrupt and wayward transitions (table 1). Transition probability should be low for both abrupt and wayward transitions, since neither type of transition is predicted to occur at the particular time it occurs, and prediction error would be elevated in both cases. (It is possible that low transition probability may be one of the prerequisites for the experience of surprise and spontaneity in thought in general.) Occurrence probability, on the other hand, should be different between abrupt and wayward transitions. The onset of an abrupt transition does not violate predictions about whether that thought content should occur in general; the implicit statistical models would already have estimated this content to have somewhat elevated (medium to high) occurrence likelihood owing to its higher local (context-specific) probability or its global baseline probability (content linked to goals, affect, motivations or the current environment). The onset of a wayward transition, by contrast, does violate predictions about whether that thought content should occur in general; this content would not be explainable based on goals, affect, motivations or current concerns. Because of this difference between wayward and abrupt transitions, we expect that the former would elicit a stronger feeling of surprise and spontaneity than the latter.

**Table 1.** Putative relationship between type of transition (abrupt versus wayward) and type of probability (transition versus occurrence). Transition probability refers to the probability that a thought *n*+1 will follow another thought *n*, and occurrence probability to the probability that a thought will occur based on its local or global baseline frequency.

	probability type	
transition type	transition	occurrence

abrupt	low	medium to high
wayward	low	low

**Figure 2.**
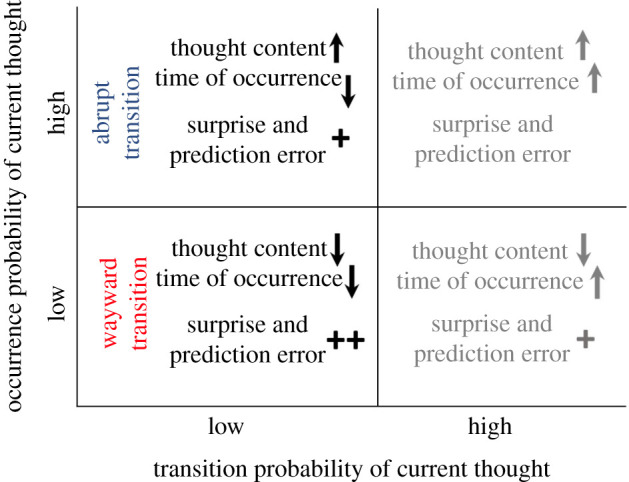
Hypothesized phenomenological differences related to surprise and prediction error in thought predictions for different levels of transition and occurrence probabilities. Upwards and downwards arrows indicate high and low expected probability, respectively. The hypothesized level of surprise and prediction error are indicated with 0, 1 or 2 plus (+) signs. Greyed out areas are mostly beyond the scope of this paper but are valuable subjects for future work. (Online version in colour.)

We also hypothesize that there would be specific phenomenological differences related to surprise and prediction error for different levels of transition and occurrence probabilities, illustrated in figure 2. The present paper focuses on wayward and abrupt transitions, both of which have relatively low transition probability. Although thought transitions with relatively high transition probability are largely beyond the scope of this paper, they are worth a brief mention. The upper-right quadrant in figure 2, with high transitional and high occurrence probability, describes conditions during which thoughts will produce little to no surprise or prediction error—perhaps the more ‘mundane’ thoughts we experience during the day or the relatively routine goal-directed problem solving we engage in. The lower-right quadrant, on the other hand, with high transition and low occurrence probability, suggests an intriguing type of thought transition where the time of transition is not suprising, but thought content is surprising. Such thought transitions may occur during states of flow [36], or dreaming [37], an important topic for future investigation that is unfortunately beyond the scope of this paper.

### (d) Surprising thoughts: a glitch or a feature?

Why do surprising transitions in thoughts occur at all? If the brain is principally concerned with reducing surprise and prediction errors, as some predictive processing accounts of brain function have proposed [18,38], it might appear maladaptive for the brain to produce thought transitions that increase rather than decrease surprise and prediction errors. The occurrence of surprise-inducing transitions in thought may appear to pose a challenge to traditional predictive processing accounts. One answer to that challenge is to regard such thoughts as a glitch in the brain's prediction error minimization functions. We suggest, however, that such surprising transitions are instead an important and highly adaptive feature of our thought streams. In our view, such thought transitions bespeak the importance of novelty seeking for predictive systems [22,39]. Novelty-seeking behaviours are essential for learning and development, starting in infancy [40,41]. The generation of thoughts that violate our implicit statistical predictions are likely an adaptive mechanism for testing and improving our predictive models through exploring hypothetical prediction errors and proactively adapting the models to account for them. This appears to occur through the use of mental simulations that do not rely upon immediate sensory stimuli, and may even be enhanced by the attenuation or cessation of incoming sensory signals (such as the blocking of incoming sensory signals that occurs during rapid eye movement (REM) sleep [37]). This process could potentially reduce future prediction errors above and beyond what prediction errors based on immediate sensory signals can do. Abrupt and wayward transitions may confer distinct benefits along these lines.

#### (i) Abrupt and wayward transitions: potentially distinct benefits

Abrupt transitions tend to bring about thoughts related to current concerns or unresolved goals that an organism has yet to attain. Thus, abrupt transitions may draw attention to unresolved goals by preferentially re-orienting the organism's attention to content that is relevant to such goals, thereby increasing the chance that they will be attained over time. This notion aligns well with the idea that animals, including humans, often engage in exploratory behaviours to help with goal fulfilment over time [42]. Abrupt transitions could be an example of such exploratory behaviour.

Wayward transitions, on the other hand, are likely related, at least in part, to novelty seeking. Although both types of transitions may be linked to novel mental simulations, wayward transitions may lead to more spatio-temporally distant episodic simulations or involuntary semantic memories [15]. These variations in our typical thought stream may help us extract important features from our experience to improve our understanding of the world through creating conditions of interleaved learning [43] below the level of conscious awareness [44].

Wayward transitions may also be beneficial for updating high-level features of the statistical model by broadening the potential mental search space to include contents that may otherwise be neglected owing to their low baseline occurrence probability. For example, involuntary memories about rare occasions of sleep deprivation may be useful for updating models about the self or incorporating past experience into new decisions [45]. Wayward transitions in thought may stem from mechanisms similar to those that drive novelty-seeking behaviours [40,41]. We are able to learn from novelty even when it is only simulated in our thoughts, and we may even have evolved to welcome and enjoy such novel thoughts, given the close association between novelty seeking and dopamine [46,47].

## 3. Possible neural correlates of abrupt and wayward transitions

**Figure 3.**
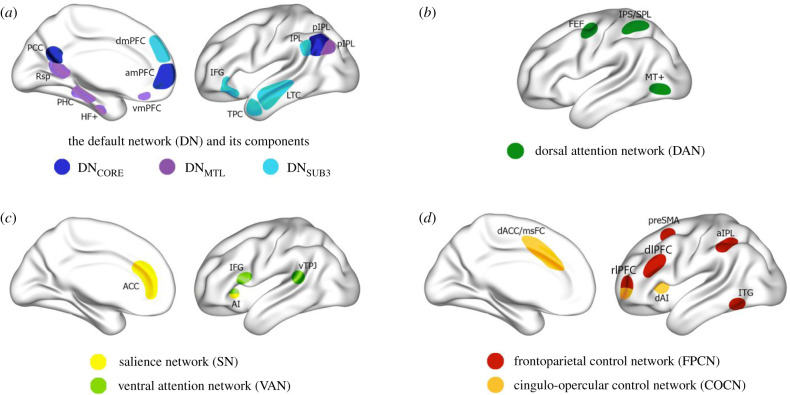
Large-scale brain networks with importance for spontaneous thought. Adapted from Christoff *et al*. [5]. (*a*) The default network (DN) is centred on the medial prefrontal cortex (mPFC), the medial parietal cortex and the lateral parietal cortex and extends into the temporal lobe and lateral PFC. Three subcomponents within the DN have been identified: (i) DN_CORE_ includes the anterior mPFC (amPFC), posterior cingulate cortex (PCC) and posterior inferior parietal lobule (pIPL), (ii) DN_MTL_ includes the hippocampal formation (HF), parahippocampal cortex (PHC) and a number of medial temporal lobe cortical projections, such as the retrosplenial cortex (Rsp), the ventral mPFC (vmPFC) and the pIPL, (iii) DN_SUB3_ extends more dorsally and includes the dorsomedial PFC (dmPFC), the lateral temporal cortex (LTC) extending into the temporopolar cortex (TPC) and parts of the inferior frontal gyrus (IFG). All three DN subsystems seem to include subsections of the IPL. (*b*) The dorsal attention network (DAN) comprises a distributed set of regions centred around the intraparietal sulcus (IPS)–superior parietal lobule (SPL), the dorsal frontal cortex along the precentral sulcus near, or at, the frontal eye field (FEF) and the middle temporal motion complex (MT+). (*c*) The ventral attention network (VAN) comprises a ventral frontal cluster of regions, including the inferior frontal gyrus (IFG), the anterior insula (AI) and the adjacent frontal operculum (not shown); it includes the ventral temporoparietal junction (vTPJ). Although the VAN is predominantly right lateralized, a bilateral salience network (SN) has also been defined. The most prominent regions of the SN are the AI and the anterior cingulate cortex (ACC). (*d*) Two ‘control’ networks have been discussed in the literature. The frontoparietal control network (FPCN) includes the dorsolateral PFC (dlPFC) and the anterior IPL (aIPL). Under a broader definition, the FPCN extends to regions including the rostrolateral PFC (rlPFC), the region anterior to the supplementary motor area ((pre)SMA) and the inferior temporal gyrus (ITG). The cingulo-opercular control network (COCN) includes the dorsal ACC (dACC)–medial superior frontal cortex (msFC) and bilateral AI–frontal operculum. The rlPFC contributes to both the FPCN and COCN. Not every network illustrated here is discussed in the present paper. (Online version in colour.)

So far, we have suggested that abrupt and wayward transitions are distinguishable based on phenomenology, probability metrics and their potential benefits. Can they also be distinguished in terms of their underlying neural correlates? Neuroimaging research has shown that spontaneously arising thoughts are precipitated by neural activity across specific brain networks, including the hippocampus and parahippocampus [4]. According to the Dynamic Framework of Thought [5], such thoughts may arise through the interactions of multiple large-scale brain networks (figure 3). Below we provide some specific hypotheses as to what the similarities and differences between wayward and abrupt transitions at the neural level may be, based on previous empirical findings and theoretical work, including the Dynamic Framework of Thought [5].

Abrupt and wayward transitions share some phenomenological properties, such as a lack of deliberate generation and surprise at a thought content arising unexpectedly with respect to its time of arising. They likely also share a relatively sharp change in thought content relative to the immediately preceding content. These shared phenomenological properties likely correspond to at least some partially shared neural correlates. An absence of deliberate generation is likely to correlate with decreased recruitment and functional connectivity within the frontoparietal control network (FPCN), given the FPCN's well-established role in voluntary cognitive control [48,49]. A sharp change in thought content may coincide with relatively rapid alterations in dynamic functional connectivity within the core component of the default network (DN_CORE_), a network that has been linked to the maintenance of high-level representations of information [27], including ongoing thought content [50]. Such higher-order representations of ongoing thought content would require updating during abrupt and wayward transitions, and these changes will likely be reflected in dynamic alterations of functional connectivity within the DN_CORE_ itself. The medial temporal lobe component of the default network (DN_MTL_) is also likely to be involved in any sharp change in thought content, given that nodes of this subnetwork such as the hippocampus appear to be a reliable internal source of spontaneously arising thought content [4,5,44]. The involvement of the hippocampus in sharp content transitions may also be evident through increases in its associated theta rhythm power, which partially indexes the semantic distance between contiguously recalled items [51].

In the light of their phenomenological divergences, abrupt and wayward transitions are also likely to exhibit some distinct neural correlates. Abrupt transitions are brought about by current goals, implicit affect, motivations or current concerns. Because of this, abrupt transitions may be associated with increased salience network (SN) and ventral attentional network (VAN) recruitment and precedence of influence on overall brain dynamics, given these networks' involvement in assigning, detecting and initiating attentional orientations based on stimulus salience [52,53]. Abrupt transitions may also be associated with increased recruitment of the ventromedial prefrontal cortex and anterior hippocampus, owing to these brain regions' involvement in constructing future-oriented simulations that rely on prior knowledge, which may be used to fulfil goals or address current concerns [50,54].

Wayward transitions, on the other hand, do not appear to be linked to any obvious latent emotions, motivations, goals or concerns. Unlike abrupt transitions then, wayward transitions are unlikely to be associated with strong recruitment and functional connectivity within the FPCN, SN and VAN, all of which are known to mediate explicit or latent motivations, goals and concerns. Instead, wayward transitions may be linked to the generation of relatively novel content in the thought stream—content that would be experienced as novel both in terms of its transition and in terms of its occurrence probability. Wayward transitions, therefore, may be associated with greater recruitment and precedence over brain dynamics of the DN_MTL_, owing to this network's role in generating novel thought content [6,55] and detecting and signalling novelty [56–58].

It is also likely that there are specific neural correlates underlying the computation of predictions, prediction errors, and transitional and occurrence probabilities in the thought stream. Current neuroscientific knowledge does not lend itself to making specific neural predictions in relation to these predictive processing mechanisms, but future empirical and theoretical investigations of these mechanisms will play a crucial role in expanding our scientific knowledge of surprise and spontaneity in the thought stream.

## 4. Conclusion

Thoughts that seem to come ‘out of the blue’ or ‘out of nowhere’ have puzzled us for millennia. For nearly all of human history, such thoughts—especially the most sudden, insightful and important—were almost universally attributed to the divine or other external spiritual sources. The modern and seemingly obvious view that they come from one's own mind or brain is in fact an incredibly recent perspective on their sources [59]. Scientific understanding of spontaneous thought has progressed by leaps and bounds in recent years, but we still lack a comprehensive account of the phenomenology of this peculiar kind of mental spontaneity and of how it relates to the neural processes that give rise to it. In this paper, we have focused on two different phenomenologically distinct forms of spontaneity in thought: abrupt and wayward transitions. Mental spontaneity, however, is by no means a unitary phenomenon and in addition to abrupt and wayward transitions, there are numerous other phenomenologically, probabilistically and neurally distinct aspects of spontaneity that are important subjects for future work and discussion.

Our scientific understanding of human thought in general, and the phenomenology of mental spontaneity in particular, will benefit enormously from the expanded use of predictive processing and probabilistic principles as explanatory tools in our scientific theories. Predictive processing accounts of thought have not yet become mainstream in the scientific literature. Their development and wide-spread adoption could, however, be a crucial stepping stone towards reaching a place of clearer understanding about the origins of spontaneous thoughts. Given the enormous role that spontaneous thought plays in individual mental health outcomes [5,60] and societal wellbeing more broadly, reaching that place is of great importance.

## Endnotes

1 We use the terms *abrupt* and *wayward* in an attempt to mirror some of the distinct phenomenological characteristics associated with these two types of thought transitions.

## Data accessibility

This article has no additional data.
